# Sodium fluoride induces skeletal muscle atrophy via changes in mitochondrial and sarcomeric proteomes

**DOI:** 10.1371/journal.pone.0279261

**Published:** 2022-12-22

**Authors:** Apoorva H. Nagendra, Animikh Ray, Debajit Chaudhury, Akash Mitra, Anu Vinod Ranade, Bipasha Bose, Sudheer Shenoy P.

**Affiliations:** 1 Stem Cells and Regenerative Medicine Centre, Yenepoya Research Centre, Yenepoya Deemed to be University, Deralakatte, Mangalore, India; 2 Father Muller Research Centre, Father Muller Medical College, Father Muller Charitable Institutions, Kankanady, Mangalore, Karnataka, India; 3 Department of Basic Medical Sciences, College of Medicine, University of Sharjah, Sharjah, United Arab Emirates; University of Montreal, CANADA

## Abstract

Sodium Fluoride (NaF) can change the expression of skeletal muscle proteins. Since skeletal muscle is rich in mitochondrial and contractile (sarcomeric) proteins, these proteins are sensitive to the effects of NaF, and the changes are dose-and time-dependent. In the current study, we have analysed the effect of high concentrations of NaF (80ppm) on mouse skeletal muscle at two different time points, i.e., 15 days and 60 days. At the end of the experimental time, the animals were sacrificed, skeletal muscles were isolated, and proteins were extracted and subjected to bioinformatic (Mass Spectrometric) analysis. The results were analysed based on changes in different mitochondrial complexes, contractile (sarcomeric) proteins, 26S proteasome, and ubiquitin-proteasome pathway. The results showed that the mitochondrial proteins of complex I, II, III, IV and V were differentially regulated in the groups treated with 80ppm of NaF for 15 days and 60 days. The network analysis indicated more changes in mitochondrial proteins in the group treated with the higher dose for 15 days rather than 60 days. Furthermore, differential expression of (sarcomeric) proteins, downregulation of 26S proteasome subunits, and differential expression in proteins related to the ubiquitin-proteasome pathway lead to muscle atrophy. The differential expression might be due to the adaptative mechanism to counteract the deleterious effects of NaF on energy metabolism. Data are available via ProteomeXchange with identifier PXD035014.

## Introduction

Fluoride (F) ameliorates dental caries through drinking water and fluoride-containing products [1]. However, excessive fluoride ingestion can lead to fluorosis and toxicity to the musculoskeletal system [2] and tooth [3]. Population residing around fluoride endemic areas worldwide suffer from bone and muscle weakness [2]. Skeletal fluorosis is characterized by the thickening of the periosteum, calcification of muscle, tendons, ligaments, and multiple hypertrophic bony projections near the ligament and atrophic muscle attachments to bone [4]. Along with bone, muscles get affected by fluorosis, and muscles change (hypertrophy and atrophy) depending upon the fluoride dose and exposure period [5–10].

Skeletal muscle mass depends upon the rate of protein synthesis and protein degradation. It can increase its mass, i.e., hypertrophy during development, exercise, stretch, mechanical loading and blocking myostatin (GDF-8) [11]. Similarly, muscle mass can be reduced (muscle atrophy) with an increase in protein degradation observed during ageing, disuse, hindlimb suspension, glucocorticoid (dexamethasone) treatment, and COPD [12–15]. However, most proteins are degraded by the ubiquitin-proteasome system (UPS) through activated FoxO transcription factors inducing the expression of Atrogin-1 and MuRF1 [16–18]. UPS degrades myofibrillar proteins by ATP-dependent polyubiquitination and subsequent degradation in the 26S proteasome. Polyubiquitination of proteins requires three different ubiquitin enzymes, the E1, E2 and E3 ligases. Some E3 ligases are muscle-specific and catalyse the final step in the ubiquitination cascade, granting its specificity to the UPS [19–21]. Downregulation of E3 ligases disrupts processes and pathways relevant to mitochondria and their function, including the TCA cycle, ETC and Fatty acid oxidation [22]. Intake of fluoride in drinking water causes alterations in mitochondrial function and aerobic respiration in the inner mitochondrial membrane and has a critical role in oxidative stress, apoptosis and cell proliferation [8,9,23–29].

We hypothesized that short-term and long-term fluoride exposure could lead to skeletal muscle atrophy via activating UPS. Though many studies are done on UPS and muscle atrophy, a detailed analysis of reduction in mitochondrial activity causing activation of proteolytic pathways leading to skeletal muscle atrophy is lacking. We have investigated the effects of NaF on mitochondrial activity and activation of UPS (muscle-specific E3 ubiquitin ligase) by label-free proteomics on the mouse muscles exposed to water containing high concentrations of NaF and characterized the altered proteins.

## Material methods

Animals C57BL/6 mice five weeks old were purchased from Adita Biosystems Private Limited Karnataka, India: 1868/PO/Bt/S/16/CPCSEA), a certified animal vendor. The animals were maintained in the individually ventilated caging (IVC) system in the animal house throughout the experiment at controlled humidity, 12h light/dark cycle at 25°C temperature. All animal experiments were carried out according to the Institutional animal ethical committee (IAEC) guidelines of Yenepoya (Deemed to be University) (347/PO/Re-S/Rc-L/01/CPCSEA), Mangalore, prior to the study.

### Experimental design

Mice were divided into four groups. The control group was given normal drinking water, and the treated group was given 80 ppm NaF dissolved in drinking water for 15 and 60 days. Water consumed by the mice was measured and changed every day. The weights of the mouse were measured once a week. At the end of each time point mouse was euthanized by CO_2_ **asphyxiation,** and hind limb skeletal muscles were harvested for further experiments.

### Protein isolation and digestion

For proteomics analysis, 20mg of skeletal muscle tissue was taken. Isolated tissues were washed thrice in ice-cold 1×PBS and with lysis buffer (1mM sodium orthovanadate, 2.5mM sodium pyrophosphate, 1mM β glycerol phosphate in 50 mM TEABC, and 4% SDS). Lysates were sonicated on ice using Q-Sonica (Cole-Parmer, India), followed by centrifuging at 13,000g for 30min, and the supernatant was taken for further processing. Protein concentration was estimated using the bicinchoninic acid (BCA) assay kit and was also confirmed visually resolving on a 10% SDS-PAGE gel. Based on the protein concentrations, 1mg of protein was processed for each sample.

### Trypsin digestion and peptide fractionation

Protein (1mg) was reduced with DTT and alkylated with iodoacetamide. 50mM of triethylammonium bicarbonate buffer (TEABC) was added to decrease the molarity of urea to 1mM, followed by digestion with trypsin overnight at a ratio of 1:20 of enzyme/protein at 37°C. Peptide desalting was carried out with the Stage Tip method, as described previously [30]. The C-18 material was stacked onto 200 μL tips, activated with 100% ACN, and equilibrated with 0.1% formic acid. The sample was passed twice, washed with 0.1% formic acid and elution with 40% ACN in 0.1% formic acid. The dried fractions were stored at -20°C until LC-MS/MS analysis.

### Mass spectrometry and proteomic data acquisition

Proteomic data were acquired using a High-Resolution Liquid Chromatograph Mass Spectrometer (Orbitrap, Thermo Fischer Scientific, Bremen, Germany) connected to Easy-nLC-1200nanoflow liquid chromatography (Thermo Fisher Scientific). Briefly, the peptides were reconstituted and quantified using a peptide assay kit and loaded onto a trap column. Full MS scans were done (350–1800 m/z) at a resolution of 70000 with a target value of 1.00 + E6 and an allowed ion accumulation time of 60 min. Data for the *in vivo* sets were acquired in triplicates. The mass spectrometry proteomics data have been deposited to the ProteomeXchange Consortium via the PRIDE partner repository with the dataset identifier PXD035014 [31–33].

### Protein identification and data analysis

Proteomill was deployed for protein identification and data analysis as a web-based application that uses a shiny server to execute the logic at the server-side end of the application. Amazon web services are the host of ProteoMill. The architecture of the software is designed in R (version 3.6.1). The R-package shiny [34] and shiny dashboard version 0.7.1 [35] were used to design the application interface using a customized CSS theme. Animated features were designed with **jQuery** and the **Anime.js** library. The plots were generated using several standard R packages like **ggplot2 [36]**, **Heatmaply** [37] for the creation of heatmaps, **networkD3 [38]** and **vis Network** for the generation of network maps.

### Bioinformatics analysis

Proteomill performed principal component and multilevel principal component analysis for data quality control using the R-package stats and a different package **mixOmics** that exclusively performs the multilevel principal component analysis. Proteomill performs differential expression analysis using R-packages like **limma** [39] and **DESeq2 [40]**. Two contrasts were identified, and a paired or non-paired design was selected to perform differential expression analysis. The default setting of the ProteoMill software was selected to analyze the proteomic dataset to assess the significance of differential expression. In order to perform functional enrichment and network analysis, pathway and interaction data are collected dynamically from the **Reactome** database [41] and **STRING** database [42]. Data analysis for pathway and interaction studies was obtained by the ProteoMill software from Reactome [41] and **STRING** platforms [42].

### Quantitative real time-PCR (RT-PCR)

Total RNA was extracted from tissue, and C2C12 myoblasts were treated with or without NaF using RNeasy mini kit (Qiagen 74104). cDNA was reverse transcribed using iScript RT (Bio-Rad 1708891), and subsequently, quantitative PCR was performed using SsoFast^TM^ Eva Green super mix (Bio-Rad 1725201), as per manufacturer’s instructions. Gene expression was carried out for various markers related to Mitochondrial complex-1, E3 Ubiquitin ligases and 26S proteasome. Gene expression levels were normalized to GAPDH, with relative gene expression fold-change calculated using the 2^(-ΔΔCt)^ method. The list of primers used can be found in S1 Table.

### Proteasome activity assay

Cell lysates were prepared from C2C12 cells treated with different concentrations of NaF (1.5PPM and 5PPM). Proteasome activity were performed using a proteasome activity assay kit (ab107921, Abcam) according to the manufacturer’s protocols. The chymotrypsin-like activity was measured using an AMC-tagged peptide substrate Proteasome Substrate (Succ-LLVY-AMC in DMSO), which releases free, highly fluorescent AMC in the presence of proteolytic activity. The Fluorescence was measured using a microplate reader with Ex/Em 350/440 nm.

### Statistical analysis

In the proteomic data all the experiments were performed in triplicates, data were analysed using Pearson’s correlation coefficient between the technical replicates. Fold change was calculated between proteins commonly identified between the 15-day and 60-day samples. Statistical analysis was performed using Student’s *t*-test. The statistical significance of the proteasome activity assay was calculated using One-way ANOVA, and the **p value <0.05 was considered significant.

## Results

### NaF treatment alters mouse TA muscle protein expression profiles

Wild-type C57BL6 mice, when treated with NaF for 15 and 60 days *in vivo*, caused a significant change in skeletal muscle proteome. In order to understand the signalling modifications possibly caused due to NaF treatment, we employed a Label-free quantification (LFQ) based quantitative proteomics approach to investigate alterations in protein expression induced by NaF during short and long-term exposure *in vivo*. An experimental flowchart for proteomic analysis is depicted in **(S1 Fig)**. In Our proteomic analysis, we have identified a total of 1231 proteins at a false discovery rate of 1%. Among 1231 proteins identified, 227 proteins were highly downregulated, 270 proteins were highly upregulated during short-term exposure to NaF, 147 proteins were significantly upregulated, and 296 proteins were significantly downregulated during long-term exposure to NaF *in vivo*. A list of totally identified and altered proteins in short and long-term exposure is provided in (**S2–S4 Tables**). The violin plots depicted in (**S2A Fig**) show the **expression levels of the proteins in each sample,** box inside shows the (logarithmized) expression levels of each treatment or sample (depending on selection). Violin plots combine box plots and kernel density plots to describe the distribution patterns of the proteins in the dataset. The Principle component analysis (PCA) plot indicated that the data from each set (control, short-term and long-term exposure) clustered together, and the NaF-treated samples had a clear separation from control (**S2A and S2B Fig**). The Pearson’s correlation analysis (heat map) indicated a strong correlation of data between the technical replicates within the same sets while maintaining a low correlation between the 15 days (short-term) and 60 days (long-term) of NaF exposure datasets (**S2D Fig**).

### Pathway elucidation of muscle proteins obtained after NaF treatment for short and long-term exposure of mice

**Fig 1:**
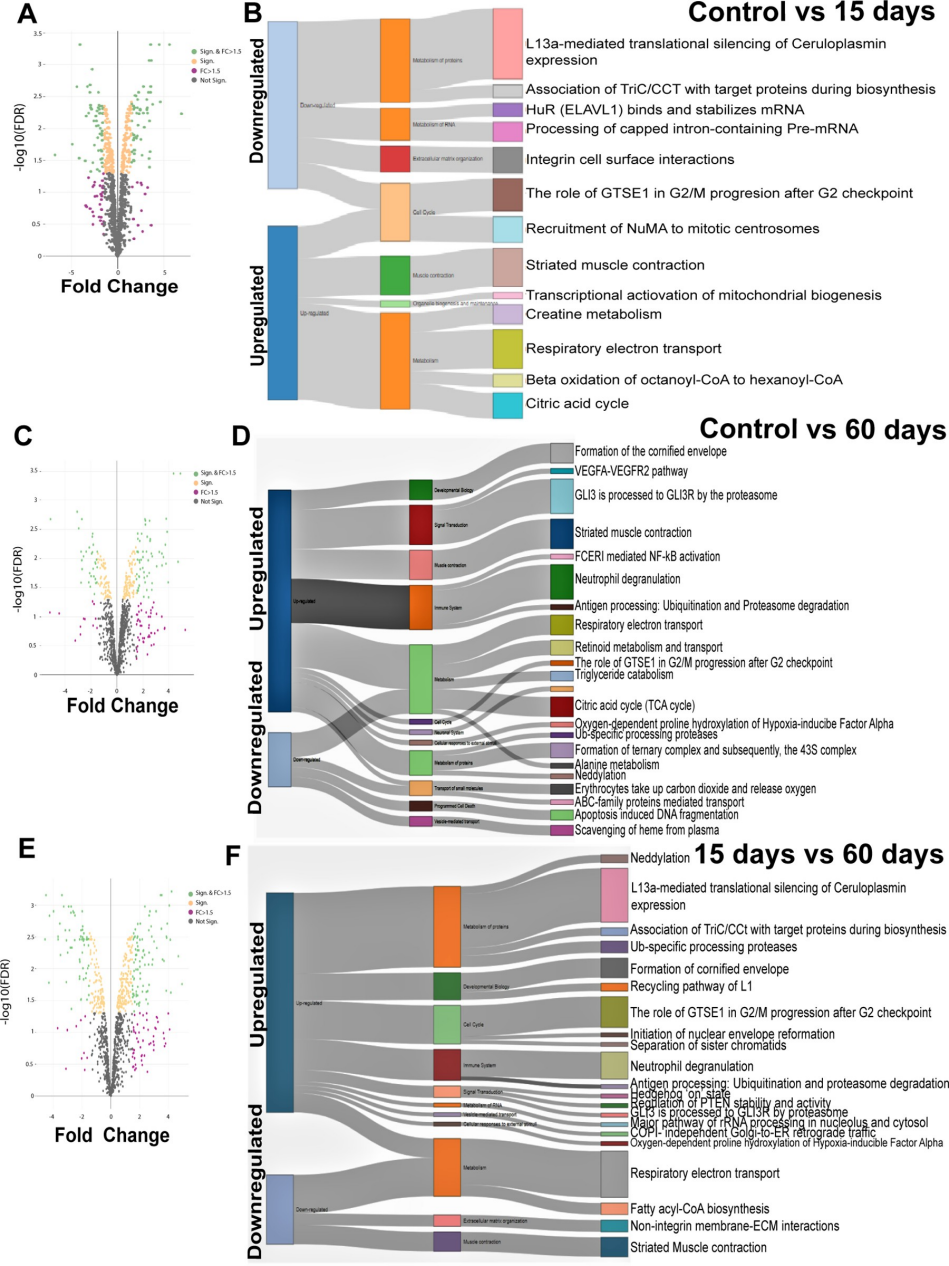
Proteome changes in different biological pathways in skeletal muscle exposed to NaF. **A & B)** Volcano plots and pathway enrichment analysis depict protein expression patterns related to specific pathway in skeletal muscle exposed to NaF for 15 days compared to control. **C & D)** Volcano plots and pathway enrichment analysis depict protein expression patterns related to specific pathway in skeletal muscle exposed to NaF for 60 days compared to control. **E & F)** Volcano plots and pathway enrichment analysis depict protein expression patterns related to specific pathway comparison in skeletal muscle exposed to NaF for 15 and 60 days.

Volcano plots depict protein expression patterns in the short (15 days) and long-term (60 days) exposure of skeletal muscle to NaF treatment. Further, out of 1420 differentially expressed proteins during short-term exposure, 550 proteins were significantly upregulated, and 592 proteins were significantly downregulated (**S2–S4 Tables**). Pathway enrichment analysis conducted for the muscle samples exposed to NaF at different time points showed upregulation and downregulation of proteins involved in different processes. The 15-day exposure to NaF indicated that proteins upregulated were related to muscle contraction (striated muscle), organelle biogenesis (mitochondria) and maintenance and metabolism (Creatine metabolism, TCA cycle, ETC and Beta oxidation). Proteins downregulated were related to the metabolism of proteins, extracellular matrix organization (integrins), and cell cycle (G2 and S Phase-Expressed Protein 1) (**Fig 1A and 1B**). Similarly, the 60-day exposure to NaF indicated that proteins upregulated were related to developmental biology, muscle contraction, Ubiquitination and proteasome degradation. Proteins downregulated were related to the cell cycle, cellular response to external stimuli and programmed cell death (**Fig 1C and 1D**). Surprisingly, when both the time points (15 and 60 days) were compared, metabolic processes such as Ubiquitination and proteasome degradation, Neddylation, Ubiquitin specific processing proteases were upregulated. Moreover, proteins of processes such as the TCA cycle, fatty acyl-CoA biosynthesis, ECM proteins and muscle contraction were downregulated (**Fig 1E and 1F**).

### Interaction network involving TCA cycle and electron transport chain proteins upon exposure to NaF

**Fig 2:**
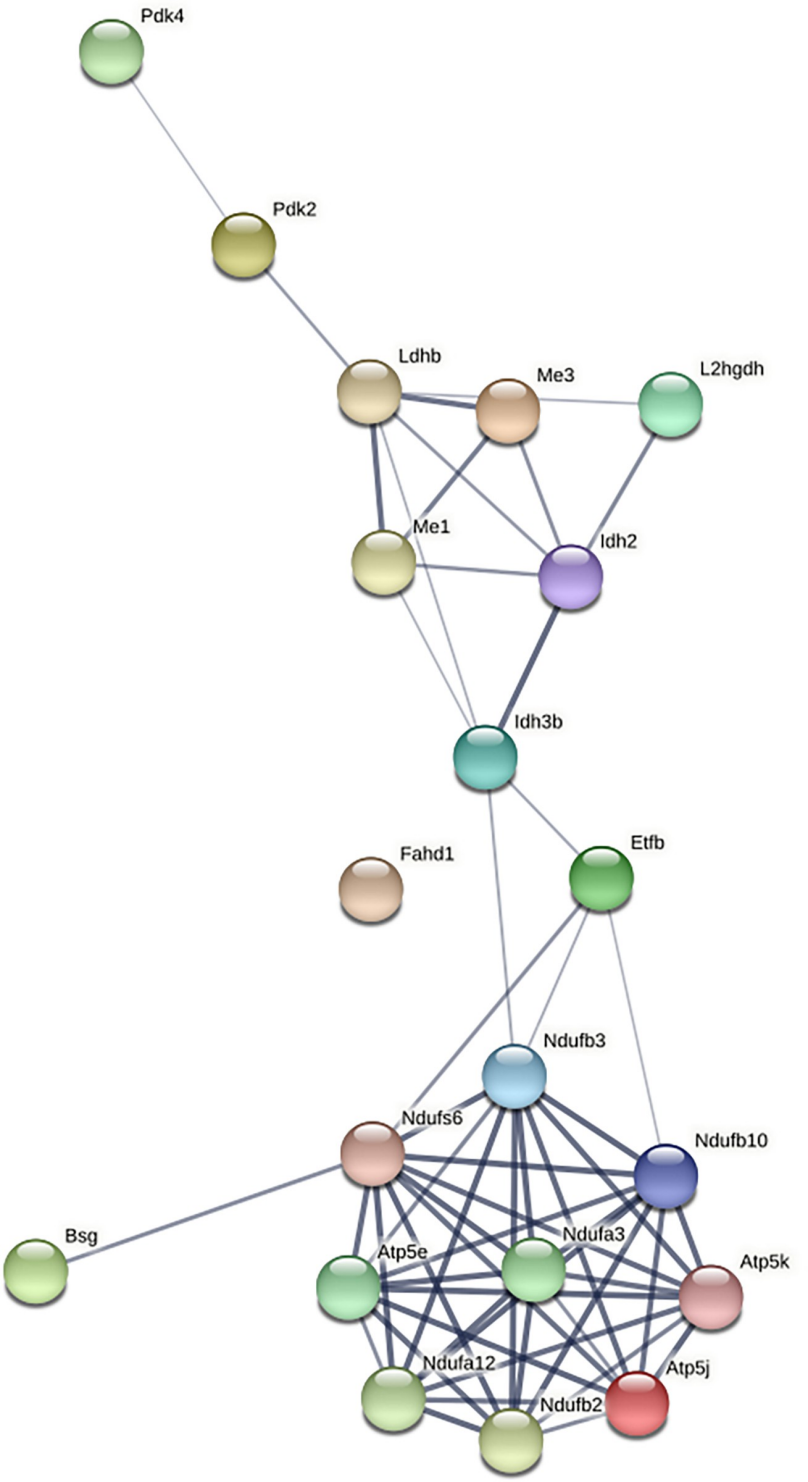
Network pathway generated by ProteoMill software to elucidate interaction of skeletal muscle proteins between TCA cycle and electron transport chain: See Table 2 for the detailed list of proteins present in the network pathway. Colour of each node indicates the differential expression of the respective protein down-regulated and up-regulated, respectively, in the treatment group (80ppm of NaF for 15 days and80ppm of NaF for 60 days vs control of each comparison).

In the interaction networks, we compared the proteins of the TCA cycle and electron transport chain (ETC) to establish a relationship between mitochondrial dysfunction and muscle atrophy. The protein interaction network indicates that when muscles were treated with 80ppm of NaF for 15 days and 60 days, most of the proteins of the TCA cycle, ETC, were downregulated. Among the altered proteins involved in energy metabolism is **Pyruvate dehydrogenase isoenzyme 4 (Pdk4-O70571)**, **L-lactate dehydrogenase (B chain Ldhb-P16125), Isocitrate dehydrogenase (P56574).** Further altered proteins related to energy metabolic (ETC) complexes such as mitochondrial **complex-I NADH: ubiquinone oxidoreductase** (**NDUFB2, NDUFA12, NDUFA3, NDUFB3, NDUFS6, NDUFB10**) were downregulated on both the periods of exposure. An Isoforms of ATP synthase (Atp5f1e- P56382) were increased during 15 days of NaF exposure, while other ATP synthase subunit gamma, mitochondrial (P97450) were decreased upon exposure to NaF for the shorter period and longer period **(Fig 2, Table 1)**. To summarize the network analysis, when the dose of 80ppm was administered for 15 and 60 days, a reduction in enzymes involved in all energetic pathways was observed (**Table 1**).

**Table 1:** Summarizes the proteins involved in network analysis (Fig 2), when the NaF dose of 80ppm was administered for 15 and 60 days, a reduction in enzymes involved in energetic pathways (ETC) was observed.

Group	Accession No.	Protein Name and Description	Gene Name	15 days Fold Change	60 days Fold Change
**ETC related proteins**	070571	Pyruvate dehydrogenase acetyl transferring kinase isozyme 4 mitochondrial	Pdk4	DOWN 0.636	NA
	P06801	NADP-dependent malic enzyme	Me1	NA	UP 1.582
	P16125	L-lactate dehydrogenase B chain	LdhB	DOWN 0.375	NA
	P18562	Bsg	Basigin	DOWN 0.669	NA
	PS2503	NADH (ubiquinone) iron -dehydrogenase sulphur protein-6, mitochondrial	**Ndufs6**	DOWN 0.405	NA
	PS4071	Isocitrate Dehydrogenase (NADP), mitochondrial	Idh2	DOWN 0.455	DOWN 0.465
	P56382	ATP synthase subunit epsilon, mitochondrial	Arp5f1e	UP 1.899	UP 2.254
	P97450	ATP synthase-coupling factor 6, mitochondrial	Atp5pf	DOWN 0.498	DOWN 0.535
	Q06189	ATP synthase subunit e, mitochondrial	Atp5me	NA	UP 1.621
	Q7TMF3	NADH dehydrogenase(ubiquinone)1 alpha subcomplex subunit 12	Ndufa12	NA	DOWN 0.622
	Q8BMF3	NADP-dependent malic enzyme, mitochondrial	Me3	NA	DOWN 0.542
	Q8R0F8	Acyl-pyruvase FAHD1, mitochondrial	Fahd1	NA	DOWN 0.379
	Q91VA7	Isocitrate dehydrogenase (NAD) subunit, mitochondrial	Idh3b	DOWN 0.622	NA
	Q91YP0	L-2-hydroxyglutarate dehydrogenase, mitochondrial	L2hgdh	NA	DOWN 0.656
	Q9CPU2	NADH dehydrogenase (ubiquinone)1 beta subcomplex subunit 2, mitochondrial	**Ndufb2**	DOWN 0.513	DOWN 0.066
	**Q9CQ91**	**NADH dehydrogenase (ubiquinone)1 alpha subcomplex subunit 3**	**Ndufa3**	**UP** **2.623**	**DOWN** **0.401**
	Q9CQZ6	NADH dehydrogenase (ubiquinone)1 beta subcomplex subunit 3	**Ndufb3**	DOWN 0.599	NA
	Q9DCS9	NADH dehydrogenase (ubiquinone)1 beta subcomplex subunit 10	Ndufb10	NA	DOWN 0.429
	Q9DCW4	Electron transfer flavoprotein subunit beta	Etfb	DOWN 0.635	
	Q9JK42	Pyruvate dehydrogenase (acetyl-transferring) kinase isozyme 2, mitochondrial	Pdk2	NA	DOWN 0.393

### Changes in mitochondrial protein related to all complexes

Mitochondria, one of the cell organelles known as the “powerhouse”, generates ATP *via* oxidative phosphorylation (OXPHOS) [43]. The inner mitochondrial membrane is made of different complexes. **Complex-1 *NADH*: *ubiquinone oxidoreductase***; **complex-II *succinate dehydrogenase***; complex**-III *ubiquinol–cytochrome c oxidoreductase (cytochrome bc1 complex)***; **complex-IV *cytochrome c oxidase*,** and **complex V*ATP synthase***.

**Fig 3:**
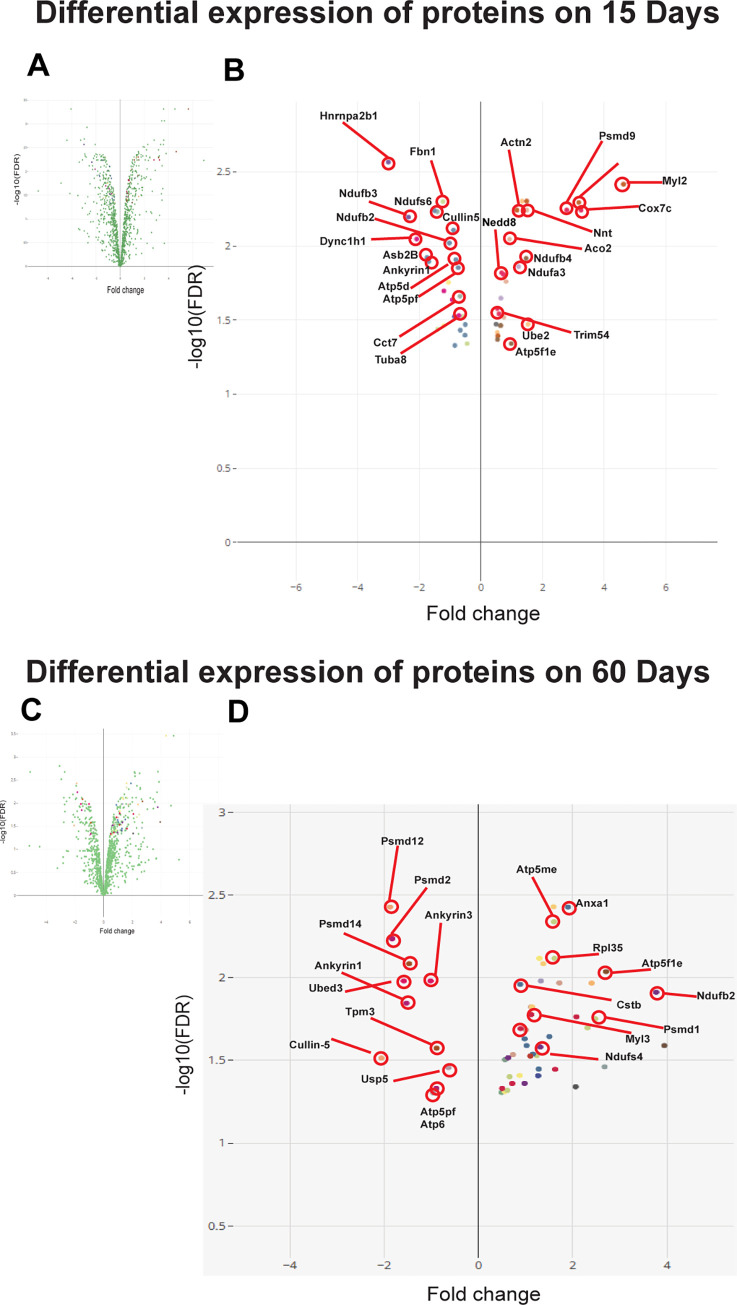
Differential expression of muscle proteins exposed to NaF 80ppm for 15 days. **A & B)** The volcano plot shows the distribution of proteins (up-regulated and down-regulated) upon short-term exposure to NaF. Differential expression of muscle proteins exposed to NaF 80ppm for 60 days. **C & D)** The volcano plot shows the distribution of proteins (up-regulated and down-regulated) upon long-term exposure to NaF.

Due to the high concentration of NaF, excess ROS is produced in the muscle myoblasts. The activity of the mitochondrial **complex-I NADH: ubiquinone oxidoreductase** (NDUFA3-2.6 fold, Q9CQ91; and NDUFB4-1.6 fold, Q4VAE8) subunits (few) were significantly upregulated upon treatment with 80ppm NaF for 15 days indicating increased mitochondrial activity (**Fig 3A and 3B, Table 2**) and (NDUFS4-1.35 fold, E9QPX3) subunit were significantly upregulated upon treatment with 80ppm NaF for 60 days with respect to control. Furthermore, the majority of the complex-1 enzyme subunits were downregulated on 15 days (NDUFB2, NDUFB3, NDUFS6, NDUFA7, NDUFB8, NDUFS8, all 0.5-fold) as well as 60 days (NDUFB2, NDUFA3, NDUFB6, NDUFA7, NDUFB10, NDUFA12, all 0.5-fold) indicating low mitochondrial activity (**Fig 3C and 3D, Table 2**). **Complex-II (succinate dehydrogenase) (Q5XK33, Sdhc)** were downregulated on both 15 days (0.5-fold) as well as 60 days (0.5-fold) (**Fig 3B and 3D, Table 2**). **Complex-III** (**cytochrome b-c1 complex)** subunit-8 **(Q9CQ69, Uqcrq; 0.5-fold)** and subunit-9 **(Q8R1I1, Uqcr10; 0.6-fold)** both were downregulated on both 15 days as well as 60 days (**Fig 3B and 3D, Table 2**). **Complex-IV**- (**cytochrome c oxidase)** all subunits **(1, 7A and 7C) Cox-1 (A0A023JC12), Cox7a21 (E9PZS8), and Cox7c (P17665)** were downregulated on 15 and 60 days (**Fig 3B and 3D, Table 2**). The activity of the mitochondrial complex-V**ATP synthase** complex is the basis for ATP production **during** oxidative phosphorylation. Subunit (**ATP5fle**-1.9 fold, **P56382**) was significantly upregulated upon treatment with 80ppm NaF for 15 days, indicating increased mitochondrial activity and (**ATP5me-1.6 fold, Q06185; ATP5fle-2.25 fold, P56382**) subunit were significantly upregulated upon treatment with 80ppm NaF for 60 days with respect to control. Further, some of the complex-V enzyme subunits were downregulated on 15 days, such as (**ATP5d, ATP5pf, ATP8**, all 0.5-fold) as well as 60 days (**ATP5, ATP6, ATP8** all 0.5-fold), indicating low mitochondrial ATP synthase activity (**Fig 3B and 3D, Table 2**).

**Table 2:** Differential regulation of proteins related to all mitochondrial complexes, sarcomeres, atrophy and Ubiquitine proteasome pathway.

Group	Accession number	Protein name and description	Gene name	15 days	60 days
**Atrophy related proteins**	Q9ERP3	Tripartite motif-containing protein 54	Trim54	UP	
	P29595	NEDD8	Nedd8	UP	
	Q922Y1	UBX domain-containing protein 1	Ubxn1	UP	
	Q561N4	Ubiquitin-conjugating enzyme E2L 3	Ube2l3	UP	
	Q9JMA1	Ubiquitin carboxyl-terminal hydrolase 14	Usp14	UP	
	Q9WV06	Ankyrin repeat domain-containing protein 2	Ankrd2	DOWN	
	G3X914	Cullin-5	Cul5	DOWN	DOWN
	P61079	Ubiquitin-conjugating enzyme E2 D3	Ube2d3		
	A0A0R4J1N7	Ankyrin-1	Ank1	DOWN	
	W6PPR4	480-kDa ankyrin G	Ank3		
	A0A0G2JGL0	Ubiquitin-conjugating enzyme E2 D3	Ube2d3	DOWN	DOWN
	P61080	Ubiquitin-conjugating enzyme E2 D1	Ube2d1		DOWN
	P56399	Ubiquitin carboxyl-terminal hydrolase 5	Usp5		DOWN
	E9QA62	Leiomodin-3	Lmod3		DOWN
**A band**	Q6ZWQ9	Myosin, light chain 12A, regulatory, non-sarcomeric	Myl12a		UP
	P09541	Myosin light chain 4	Myl4	UP	UP
	P13542	Myosin-8	Myh8	DOWN	
	Q5XKE0	Myosin-binding protein C, fast-type	Mybpc2	DOWN	
	P51667	Myosin regulatory light chain 2, ventricular/cardiac muscle isoform	Myl2	DOWN	
	Q8VCR8	Myosin light chain kinase 2, skeletal/cardiac muscle	Mylk2	DOWN	
**I band**	Q3UHZ5	Leiomodin-2	Lmod2	UP	
	Q9WV06	Ankyrin repeat domain-containing protein 2	Ankrd2	UP	
	P97447	Four and a half LIM domains protein 1	Fhl1	UP	
	Q99LM3	Smoothelin-like protein 1	Smtnl1	DOWN	
	Q9R059	Four and a half LIM domains protein 3	Fhl3	DOWN	
	P49813	Tropomodulin-1	Tmod1	DOWN	
	P09542	Myosin light chain 3	Myl3	DOWN	
**M band**	A2ABU4	Myomesin-3	Myom3	UP	
	A2ASS6	Titin	Ttn	DOWN	
	Q62234	Myomesin-1	Myom1	DOWN	
	Q14BI5	Myomesin 2	Myom2	DOWN	
	P07310	Creatine kinase M-type	Ckm	DOWN	
**Z band**	P50462	Cysteine and glycine-rich protein 3	Csrp3	UP	
	P14602	Heat shock protein beta-1	Hspb1	UP	
	O88990	Alpha-actinin-3	Actn3	DOWN	
	Q9ET54	Palladin	Palld	DOWN	
	Q8R4E4	Myozenin-3	Myoz3	DOWN	
	O70209	PDZ and LIM domain protein 3	Pdlim3	UP	
	P10605	Cathepsin B	Ctsb		DOWN
	Q8BRK8	5’-AMP-activated protein kinase catalytic subunit alpha-2	Prkaa2		DOWN
	P10605	Cathepsin B	Ctsb		DOWN
**Complex I**	Q9CQC7	NADH dehydrogenase [ubiquinone] 1 beta subcomplex subunit 4	Ndufb4	UP	
	Q9CQ91	NADH dehydrogenase [ubiquinone] 1 alpha subcomplex subunit 3	Ndufa3	UP	DOWN
	P52503	NADH dehydrogenase [ubiquinone] iron-sulfur protein 6, mitochondrial	Ndufs6	DOWN	
	Q9CPU2	NADH dehydrogenase [ubiquinone] 1 beta subcomplex subunit 2, mitochondrial	Ndufb2	DOWN	DOWN
	Q9Z1P6	NADH dehydrogenase [ubiquinone] 1 alpha subcomplex subunit 7	Ndufa7	DOWN	DOWN
	Q9D6J5	NADH dehydrogenase [ubiquinone] 1 beta subcomplex subunit 8, mitochondrial	Ndufb8	DOWN	
	Q9CQZ6	NADH dehydrogenase [ubiquinone] 1 beta subcomplex subunit 3	Ndufb3	DOWN	
	Q8K3J1	NADH dehydrogenase [ubiquinone] iron-sulfur protein 8, mitochondrial	Ndufs8	DOWN	
	E9QPX3	NADH dehydrogenase [ubiquinone] iron-sulfur protein 4, mitochondria	Ndufs4		UP
	Q9DCS9	NADH dehydrogenase [ubiquinone] 1 beta subcomplex subunit 10	Ndufb10		DOWN
	Q7TMF3	NADH dehydrogenase [ubiquinone] 1 alpha subcomplex subunit 12	Ndufa12		DOWN
	Q3UIU2	NADH dehydrogenase [ubiquinone] 1 beta subcomplex subunit 6	Ndufb6		DOWN
	A0A068BGR9	Complex I-B14.5a	Ndufa7	DOWN	DOWN
**Complex V**	P56382	ATP synthase subunit epsilon, mitochondrial	Atp5f1e	UP	
	Q4FK74	ATP synthase F1 subunit delta	Atp5d	DOWN	
	P97450	ATP synthase-coupling factor 6, mitochondrial	Atp5pf	DOWN	DOWN
	Q06185	ATP synthase subunit e, mitochondrial	Atp5me		UP
	P56382	ATP synthase subunit epsilon, mitochondrial	Atp5f1e		UP
	A0A023J6E9	ATP synthase subunit a	ATP6		DOWN
**Proteasome**	Q3THC1	26S proteasome non-ATPase regulatory subunit 9	Psmd9	UP	
	Q3ULG4	26S proteasome non-ATPase regulatory subunit 4	Psmd4	DOWN	DOWN
	Q3TXS7	26S proteasome non-ATPase regulatory subunit 1	Psmd1		DOWN
	O35593	26S proteasome non-ATPase regulatory subunit 14	Psmd14		DOWN
	Q9D8W5	26S proteasome non-ATPase regulatory subunit 12	Psmd12		DOWN
	Q8VDM4	26S proteasome non-ATPase regulatory subunit 2	Psmd2		DOWN
	P14685	26S proteasome non-ATPase regulatory subunit 3	Psmd3		DOWN

Overall, the activity of the mitochondrial complex I to V proteins are differentially regulated upon treatment with 80ppm of NaF for 15 days and 60 days with respect to control.

### Downregulation of sarcomeric proteins after fluoride intake and mitochondrial dysfunction

In addition to Ubiquitin proteins, sarcomeres contain proteins involved in cell signalling, gene expression control, and muscle contraction. Sarcomere contains thin and thick filaments, and MURF1 increases the breakdown of these thick filaments [44]. Most muscle-specific MyHCs, regulatory and myosin light chains (MyLCs), were downregulated after NaF treatment (Table 2). We observed that MyLC2 (P51667) and MyLC12b (Q3THE2) were significantly downregulated after NaF treatment after 60 days (Table 2). The interaction of MyLCs with MyHC proteins maintains muscle integrity and modulates the motor function of myosin, and the downregulation of these proteins leads to muscle atrophy **(Fig 3B and 3D, Table 2**).

### Changes in the muscle proteins related to skeletal muscle atrophy (Atrogenes)

Based on data analysed by the Proteomill software, we identified proteins associated with the ubiquitin processing pathway in the fluoride-treated samples for 15 and 60 days. The proteins that were upregulated on day 15 of NaF are the ubiquitin-conjugating enzyme **UBE2L3 (Q561N4),** an E2 conjugation enzyme related to endoplasmic reticulum-associated degradation (**ERAD**) [45,46], was upregulated on day 15 (fold-change den/ctrl 1.65, **Fig 3B and 3D, Table 2**). This protein interacts with the cullin-1 (**CUL1**) and F-box (**SCF**) complex and controls most of the ubiquitin-mediated protein degradation [47]. Other proteins upregulated on 15 days of treatment with NaF are E3 Ubiquitin ligase **Nedd8** (**P29595, 2.6-fold**); these E3 ligases of the MURF family are required for the rapid breakdown of sarcomeric proteins. Further, tripartite motif family proteins such as **Trim 54 (Q9ERP3)** and **Trim72 (Q6ZMU5)**, deubiquitinating enzymes **USP14 (Q9JMA1**; 1.6-fold) are significantly upregulated. Some of the E3 Ubiquitin ligases downregulated on 15 days and 60 days of NaF treatment are **UBE2d3 (A0A0G2JGL0**, 0.5-fold), **Cullin-5 (G3X914**, 0.5-fold), **Ankyrin repeat and SOCS box protein 2 (ASB2β; Q96Q27**, 0.5-fold), **Ankyrin-1 (A0A0R4J1N7**, 0.5-fold) for 15 days. In addition to the proteins mentioned above, other proteins downregulated at 60 days are **Ankyrin-3 (W6PPR4**, 0.6-fold), **USP5 (P56399**, 0.5-fold) **(Fig 3B and 3D, Table 2**)

### Changes occurring in 26S proteasome during muscle atrophy

26S proteasome catalyses the protein degradation; it comprises a core complex of 20s proteasome and is covered with 19s regulatory complexes at both ends, recognising and ubiquitinating proteins. Subunit (**PSMD9-2.1 fold, Q3THC1**) was significantly upregulated upon treatment with 80ppm NaF for 15 days, indicating increased 19s proteasomal activity with respect to control. Further, the majority of the 19s regulatory subunits of the 26S proteasome were downregulated on 15 days (**PSMD4-0.5 fold, Q3ULG4**) as well as in 60 days (**PSMD1, PSMD2, PSMD3, PSMD4, PSMD12, PSMD14** all 0.5-fold) indicating active 19s proteasomal activity **(Fig 3B and 3D, Table 2**).

### Gene expression associated with ubiquitination during NaF treatment on different days

**Fig 4:**
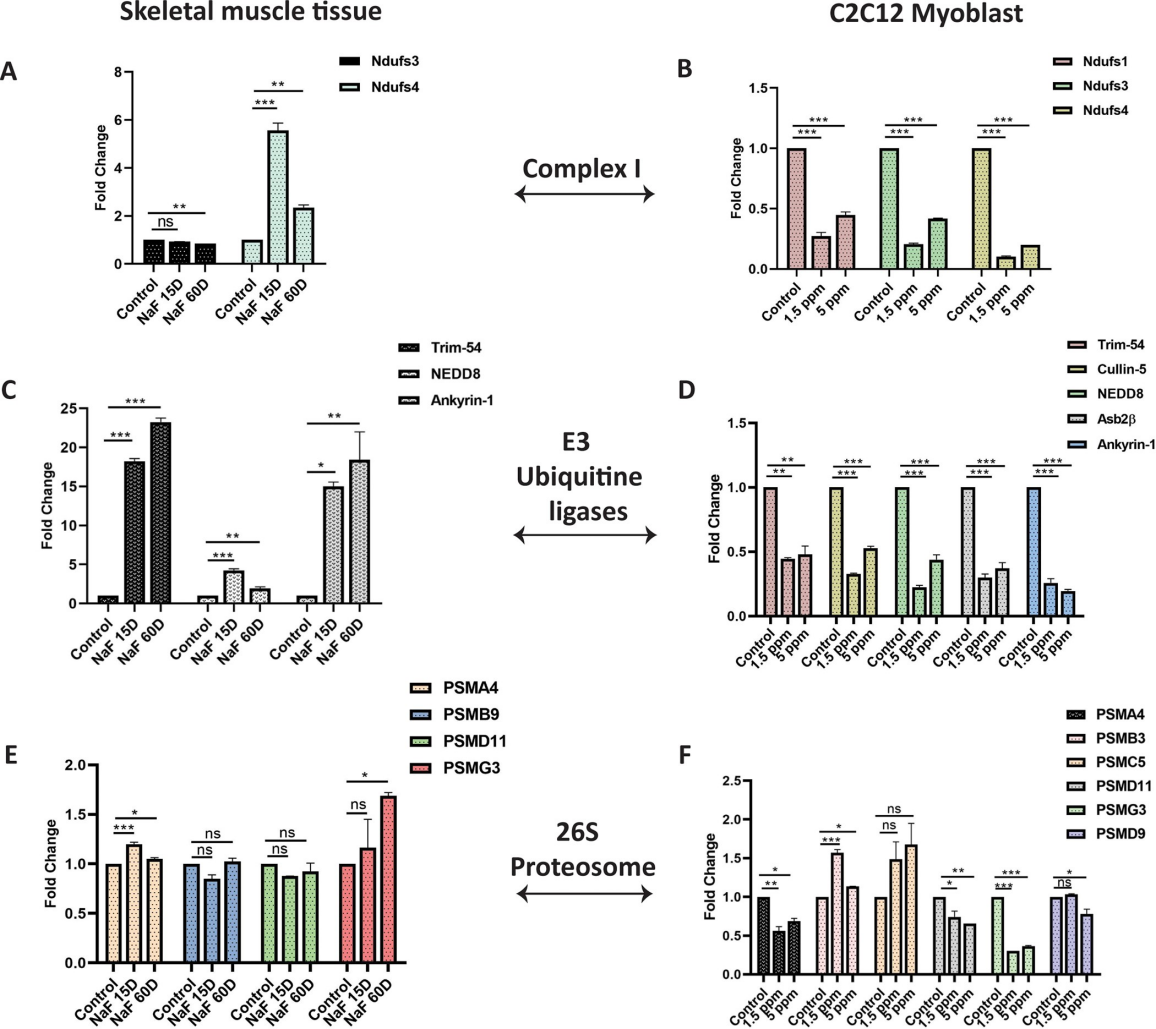
Differential Gene expression in skeletal muscle tissue and in C2C12 myoblasts after NaF treatment. **A & B**) Genes related to mitochondrial complex-1, **C & D**) E3 Ubiquitine ligase and E and F) 26S proteasome were analysed. Data are expressed as means ±SD from *n* 3/group. *P< 0.05, **P<0.01, ***P<0.001.

Gene expressions were analysed (Mitochondrial compex-1, E3 Ubiquitin ligases and 26 in the mouse TA muscle and C2C12 myoblasts by qRT-PCR at Day 15 and Day 60 of in vivo NaF treatment and 1.5ppm and 5ppm of in vitro NaF treatment. Differential gene expression was observed throughout the study in the muscle tissue and C2C12 cells. Mitochondrial complex-1 genes expressed were **NDUFS-3 and NDUFS-4** in muscle tissue. **NDUFS3** was downregulated throughout the study, but **NDUFS-4** was upregulated (5.5-fold) in 15 days of treatment compared to control. The results were consistent with protein expression observed earlier (**Fig 3A and 3B);** this might be due to increased mitochondrial activity. No gene expression was observed with other complex-1 genes, such as **ND-1 and ND-6**. **NDUFS-1, NDUFS-3, and NDUFS-4** mRNA expression was downregulated throughout the study in the C2C12 myoblast cells **(Fig 4A and 4B)**. In addition to the complex-1 subunits, the expression of other components of the ubiquitin system, i.e. (E3s, Ubiquitin ligases) was altered following NaF treatment. **TRIM-54** (17-fold at 15 days and 24-fold at 60 days), **NEDD8** (4-fold at 15 days and 2-fold at 60 days), **Ankyrin-1** (15-fold at 15 days and 17-fold at 60 days) all were upregulated in the muscle tissue in 15 days and 60 days treatment compared to control. The results of **TRIM-54 and NEDD8** were similar and comparable to protein expression in vivo. Further, most E3 Ubiquitin ligases such as **TRIM-54**, **Cullin-5, NEDD8, ASB2β and Ankyrin-1** were downregulated in vitro when C2C12 were treated with NaF, indicating the activation of these Ubiquitin ligases in the early treatment (**Fig 4C and 4D).** Analysis of the gene expression related to the 26S proteasome in the skeletal muscle tissue treated with NaF revealed that most genes related to the 19S and 20S proteasome were downregulated. Two genes related to each proteasome were analysed (**PSMD11, PSMG3 (19S), PSMA4, PSMB9 (20S)** and the results are similar and comparable to protein expression in vivo (**Fig 4E**). Similarly, when we analysed the gene expression related to 26S proteasome in the C2C12 myoblast treated with two different concentrations of NaF. One gene each from the 20S (**PSMB3**) and 19S (**PSMC5**) were upregulated. Further, all other genes (**PSMA4, PSMD11, PSMG3, PSMD9**) analysed were downregulated (**Fig 4F**).

### Proteasome activity during C2C12 myoblasts treated with NaF

**Fig 5:**
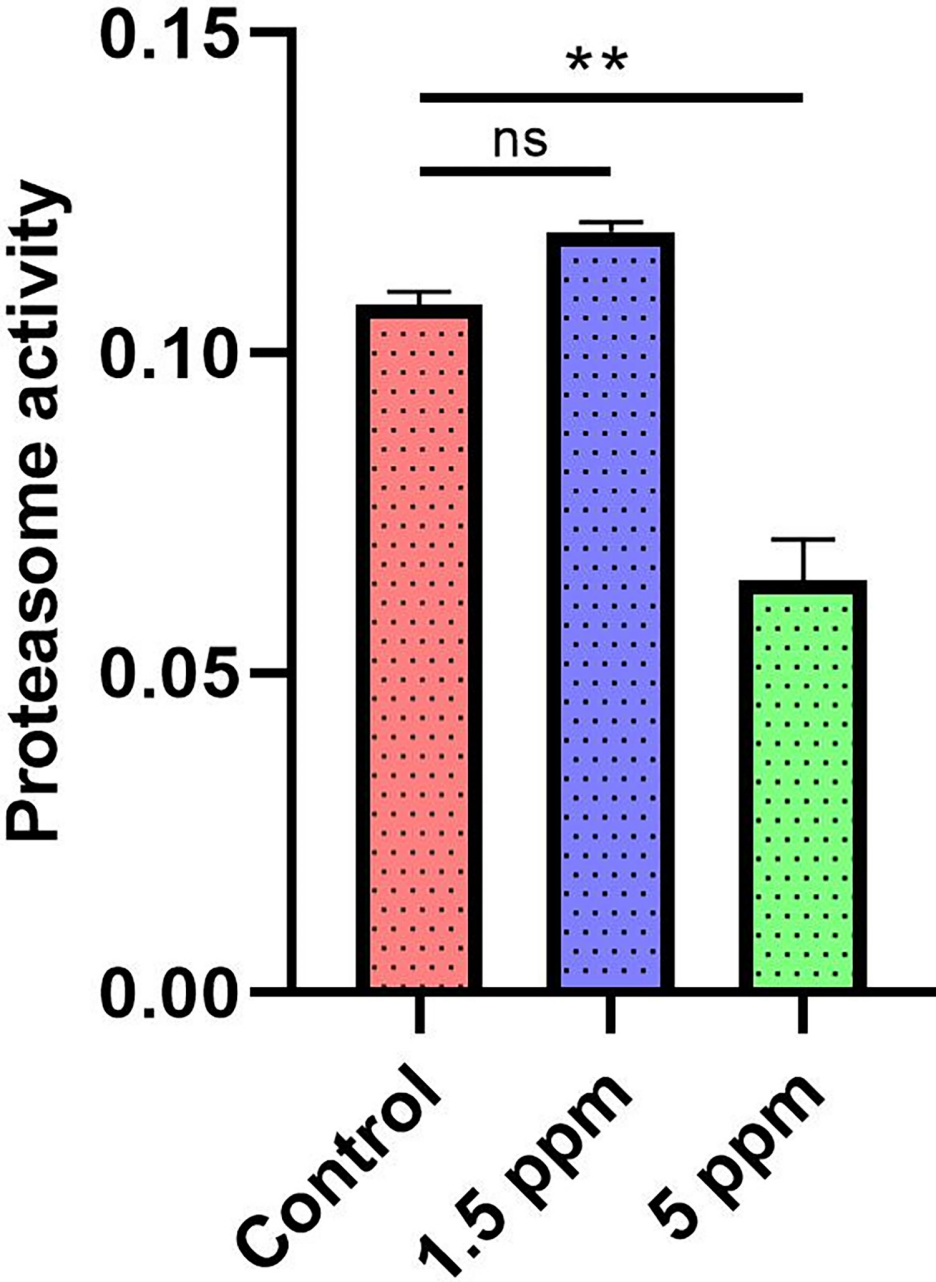
Proteasome activity in C2C12 muscle myoblasts. Chymotrypsin-like proteasome activities in C2C12 muscle myoblasts treated with different concentrations of NaF 1PPM, 1.5PPM, 5PPM compared with untreated control. Data represent means ± SEM (*p < 0.05,).

Due to the high protein turnover observed in previous experiments, we next examined the proteasome (chymotrypsin-like) activity in the C2C12 mouse myoblast cell line treated with different concentrations of NaF using the cell lysates for 24 hours. The proteasome activity or the (UPS) degrades misfolded, redundant, and damaged proteins [48]. The proteasome activity increased when treated with a low concentration of NaF (1.5PPM) (0.1132) (**Fig 5**) to the C2C12 cells when compared to the untreated control (0.1075) (**Fig 5**). Furthermore, the proteasome activity declined significantly as the concentration of NaF increased (5PPM) (0.0645) (**Fig 5**) due to the ubiquitination of the proteins.

### Protein turnover following NaF treatment

In different types of muscle atrophy, muscle protein is rapidly lost due to the suppression of protein synthesis and increased protein degradation rates [49]. A significant increase in cell proliferation and protein synthesis is seen when the skeletal muscle (mouse) was exposed to NaF (drinking water) for 15 days (80ppm concentration), causing hypertrophy of the muscle, and in the same study, we found a net protein breakdown was observed when the skeletal muscle (mouse) was exposed to NaF (drinking water) for 60 days (80ppm concentration) with an increase in protein degradation [50]. The increase in protein synthesis following NaF treatment at 15 days might be due to increased translation (mRNA) and transcriptional up-regulation following the treatment. The proteolysis activity increased due to the increase in the proteasome activity following the treatment with higher concentration and long-term exposure to NaF. Based on these observations, the protein balance has shifted to protein breakdown during 60 days of exposure with significant loss of muscle mass.

**Fig 6:**
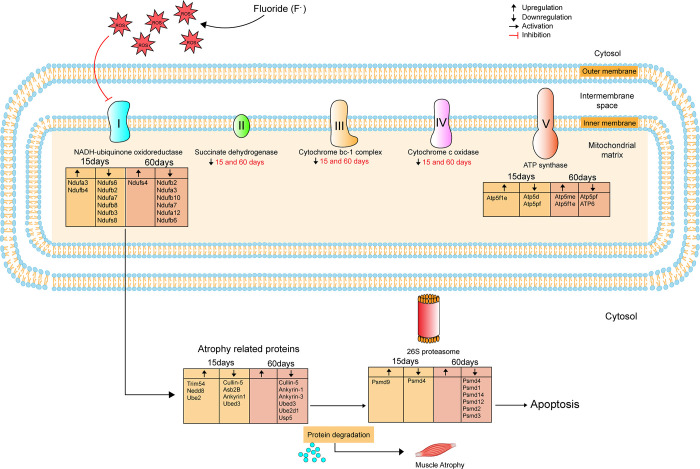
Stage wise representation of mitochondrial dysfunction and muscle atrophy caused due to ROS generated by high intake of NaF. High concentration of NaF intake for short term (15 days) and long term (60 days) causes excessive ROS production leading to inhibition of mitochondrial complexes I to V by downregulation of various complex proteins (given in the box). Inhibition of complex 1 protein causes loss of muscle proteins by the activation of atrogenes. Atrogenes activate Ubiquitin proteasome system which functions to degrade damaged myofibrillar proteins via 26S proteasome within the muscle causing muscle atrophy further leading to apoptosis.

Due to fluorosis, there is an overall change in muscle homeostasis as a function of mitochondrial protein disruption. Further, E3 Ubiquitin ligases are protein complexes that add ubiquitin molecules directly to a lysine (K48) in the amino acid side chain of a target protein or indirectly via a ubiquitin-conjugating enzyme, E2. These tagged proteins are recognized by the 19S subunit of the 26S proteasome, which delivers the target to the 20S core subunit and through its proteolytic activity that degrades the protein target [51]. On that note, most of the atrophy-related proteins mentioned in the figure are E3 ubiquitin (ub) ligases [45]. The protein product of **trim63** is MuRF1, a skeletal muscle-specific E3-ubiquitin ligase. Asb2β is another Ubiquitin-ligase with ankyrin repeats helping conjugate target proteins with the ubiquitin molecules. Cullin-5 is a recruiter of Rbx2, a RING finger protein functioning in E2 enzyme recruitment. Furthermore, in our pathway enrichment analysis, the neddylation process (post translational modification) (NEDD8) was enriched, among others which suggest a critical role in the addition of NEDD molecules (structurally and functionally similar to Ubiquitination) which triggers proteasomal destruction of muscle proteins [52] **Fig 6**.

## Discussion

Fluoride is required for the normal growth and development of the human body, but excessive fluoride concentrations can cause damage and destroy soft body tissues. Long-term exposure to fluoride results in its accumulation and can cause damage to cell organelles such as mitochondria and the endoplasmic reticulum [53]. Damages such as changes in mitochondrial membrane potential lead to its dysfunction. Further, abnormalities in mitochondrial permeability cause changes in the electron transport chain due to low ATP production by glycolysis release of cytochrome C, leading to oxidative stress and apoptosis [53,54]. In this current proteomic study, we have attempted to provide information about the effects of NaF on the skeletal muscle due to oxidative stress. Since skeletal muscle is rich in mitochondria, NaF treatment during different periods generates oxidative stress altering mitochondrial function [55].

Mitochondrial complexes I, II, and III, play an important role in regulating mitochondrial function and ATP production and are the main checkpoints for electron entry into the mitochondrial electron transport chain [56,57], where the entry and exit of the ions inside and outside the membrane take place. NaF treatment at a low or high concentration for a short period increases the mitochondrial activity of the complex-I, as shown in our study (**Fig 4, Table 1**). The initial increase in complex-1 activity might be an adaptive mechanism to improve mitochondrial respiration, resulting in enhanced ATP production and less accumulation of free radicals [58]. There is a decrease in most of the vital complex-1 subunits (NDUFB2, NDUFB3, NDUFS6, NDUFA7, NDUFB8, NDUFB10, NDUFA12) when exposed to the higher dose of NaF for a shorter and more extended period. These proteins are mainly related to mitochondrial regulation, promoting oxidation and mitochondrial ATP generation. Reducing some of these key proteins may indicate a desperate attempt to maintain balance in OXPHOS and avoid the activation of apoptotic pathways. Further, we also noticed that the activity of all the complexes decreased upon exposure to the higher dose of NaF **[59–61],** increasing the release of superoxide anions and thus increasing ROS levels [26]. The oxidative stress induced by NaF provokes the dysfunction of the mitochondrial complexes in the skeletal muscle leading to muscle atrophy.

Dysfunction of the mitochondrial complexes decreases ATP output resulting in an increased abundance of AMP. A rise in cellular AMP levels results in the activation of AMP-activated protein kinase (AMPK), which can trigger atrophic signalling by activating the transcriptional factor, FoxO3 [62,63]. Indeed, activation of AMPK in muscle fibers during prolonged inactivity is closely associated with the activation of FoxO3 [64–66]. Activation of FoxO3 promotes muscle wasting by promoting an increased expression of atrogenes involved in the ubiquitin-proteasome system and autophagy [67,68]. This activity is also observed in our current study.

The main function of the ubiquitin-proteasome system (UPS) is to degrade abnormal proteins [48]. In muscles, loss of protein by the UPS is a major mechanism involved in myofibrillar protein degradation [53]. Therefore, when we examined the proteasome activity at a low concentration of NaF (1.5ppm) on C2C12 cells, we found increased chymotrypsin-like proteasome activity, indicating that there is cell proliferation (hypertrophy during differentiation) and activation of mechanism for protein degradation. Further, as the concentration of NaF increases (5ppm), the accumulation of ubiquitinated proteins increases, indicating that proteasome-related proteolysis was enhanced. In our previous study [5,50] we saw an increase in the expression of muscle transcription factor MyoD at the same concentration (1.5ppm; low concentration and early time point) in C2C12 myoblast and C57BL6 mouse muscle was exposed to NaF. This suggests the critical role played by UPS in muscle regeneration. To extend our knowledge on alterations in the metabolism after NaF treatment, we investigated the quantitative changes in protein expression leading to muscle atrophy. The analysis revealed the activation of several pathways and proteins that might cause remodelling of the skeletal muscle proteome during NaF-induced muscle atrophy. Earlier studies have shown that several activated genes are related to muscular atrophy, known as atrogenes, associated with the catabolism of proteins. Loss of muscle proteins occurs during unloading (disuse, cancer, AIDS) [11] muscle denervation [69]. Analysis of our data sets revealed that protein expressions related to atrogenes were differentially regulated after short-term and long-term treatment with NaF.

Some of the selected atrogenes that are up or downregulated in our data sets are given in **(Table 2).** Several differentially regulated proteins participated in the ubiquitin-proteasome system (UPS) or autophagy after NaF treatment. The loss of muscle proteins mediated by activation of specific ubiquitin ligases, such as MURF1 and TRIM-32 thick filaments are mainly degraded by MURF1, and thin filament and Z-band proteins are ubiquitinated by TRIM32 [70,71]. Our study found that **MURF-1** and **TRIM 54** were involved in leading to muscle atrophy. The M-band proteins in the sarcomere are more prone to dynamic changes during early muscular atrophy [72]. In our study, M-band proteins such as myomesin-1/2, Titin-1 and Obscurin were rapidly downregulated in atrophying muscle at 15 days of exposure (**Table 2**) they were degraded by the E3 ubiquitin ligase MURF1 and TRIM54 during early atrophy. Obscurin specifically interacts with myomesin-1, which influences the stability of myomesin-1 in fast fibres [73,74]. Furthermore, we also observed an early upregulation of TRIM54 protein expression within two weeks of NaF treatment. TRIM54 is related to a group of inherited or acquired muscle disorders known as protein aggregate myopathies (PAMs), characterized morphologically by abnormal accumulation of proteins within muscle fibres [75] **TRIM54** is an atrogene encoding the muscle-specific RING finger (**MuRF**) proteins **MuRF1** and **MuRF3** (muscle-specific E3 ubiquitin ligases), respectively [76] involved in the regulation of sarcomeric protein degradation [77,78] **MURF1** and **MURF-3** have been reported to interact with several different E2 enzymes, including **UBE2K** (upregulated during short term NaF treatment), **UBE2D** and **UBE2N** (downregulated during long term NaF treatment) [47,79]. This is a strong evidence that E2 enzymes could modulate the function of E3 ligases during muscular atrophy. However, the functional relevance and mechanisms of ubiquitin chain formation still need to be determined.

## Conclusion

This innovative proteomic analysis demonstrates that marked regulation of protein synthesis, protein expression and protein ubiquitination occurs in response to the intake of high concentrations of NaF as early as 15 days (short exposure) and long as 60 days (long exposure), Further, the NaF intake showed the differential regulation of mitochondrial complexes (I-V) proteins, leading to atrophy in skeletal muscle. The high concentration of NaF suggests an attempt to reduce the energy production to fight the ROS increase and seems to be part of an adaptative mechanism of the organism to fight the deleterious effects of this ion (**Fig 6**). Additional studies evaluating the production of ATP and ROS in mitochondria upon exposure to NaF are needed to confirm the suggested adaptative mechanism. Using a label-free quantitative proteomics approach, we have identified new signalling networks and relevant signals involved in the Pathobiology of muscular atrophy.

## Supporting information

S1 Fig Schematic representation protocol adapted for identification and quantification of proteomic changes in C57BL6 mouse muscle tissues exposed to NaF for 15 days and60 days along with untreated control. (TIF)

S2 Fig Statistical analysis of proteomic data. **A)** The Violin plots combine box plots and kernel density plots depicted the **expression levels of the proteins in each sample,** box inside shows the (logarithmized) expression levels of each treatment or sample (depending on selection). **B& C)** Principal component analysis (PCA) plot indicated the data from each set (control, short-term and long-term exposure) clustered together and the NaF treated samples had a clear separation from control. **D**) The Pearson’s correlation analysis (heat map) indicated a strong correlation of data between the technical replicates within same sets while maintaining low correlation between the 15 days (short-term) and 60 days (long-term) of NaF exposure.
(TIF)

S1 Table Detail of primers that are utilized for the study. (DOCX)

S2 Table (XLSX)

S3 Table (XLSX)

S4 Table (XLSX)

S1 Graphical abstract (TIF)
