# Supplementary figures and images for: Sodium fluoride induces skeletal muscle atrophy via changes in mitochondrial and sarcomeric proteomes
Source: PLoS One. 2022 Dec 22;17(12):e0279261. doi: 10.1371/journal.pone.0279261 (PMC9779014; doi:10.1371/journal.pone.0279261)

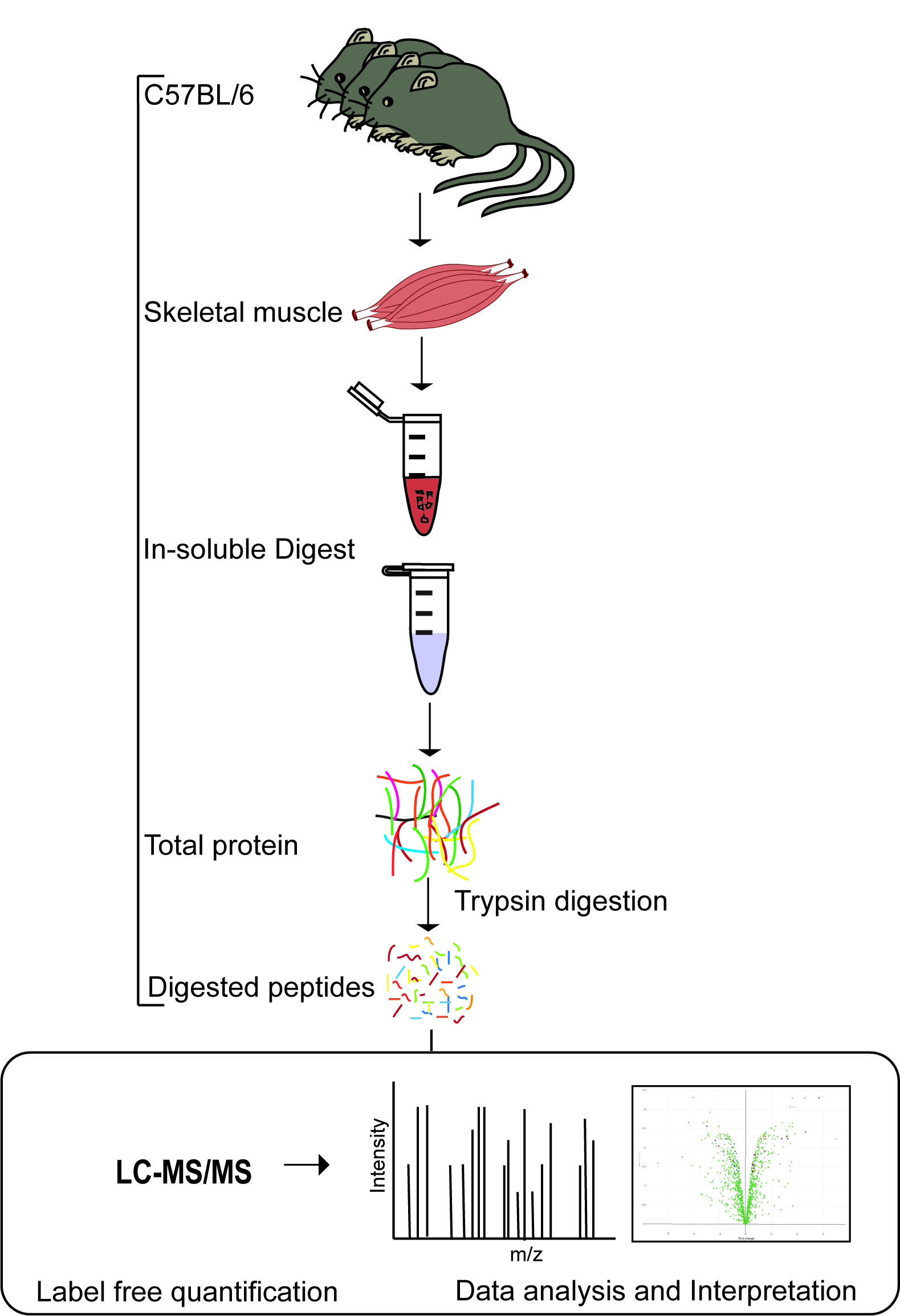

Supplement: S1 Fig — (TIF) [file pone.0279261.s001.tif]

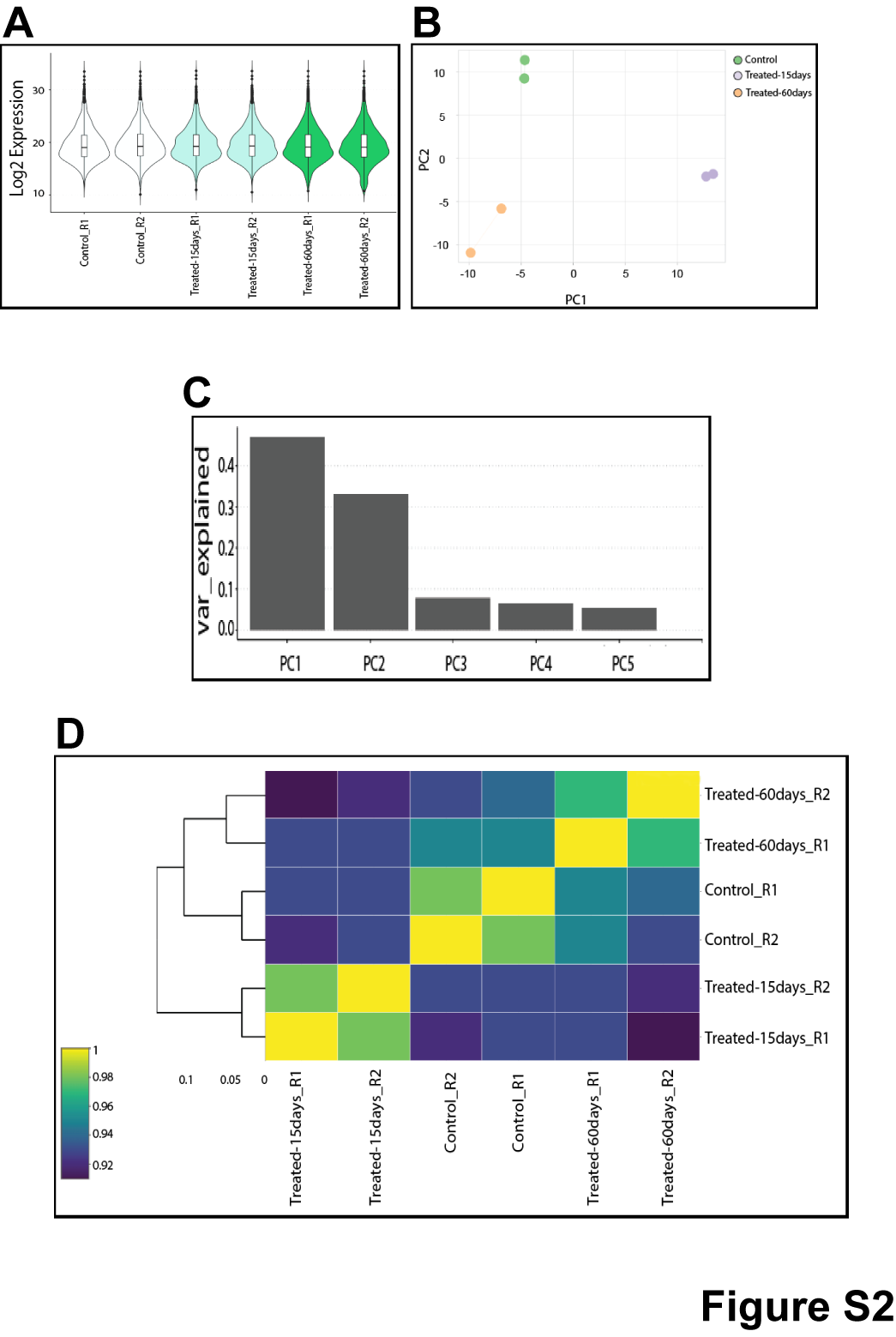

Supplement: S2 Fig — A) The Violin plots combine box plots and kernel density plots depicted the expression levels of the proteins in each sample, box inside shows the (logarithmized) expression levels of each treatment or sample (depending on selection). B& C) Principal component analysis (PCA) plot indicated the data from each set (control, short-term and long-term exposure) clustered together and the NaF treated samples had a clear separation from control. D) The Pearson’s correlation analysis (heat map) indicated a strong correlation of data between the technical replicates within same sets while maintaining low correlation between the 15 days (short-term) and 60 days (long-term) of NaF exposure. (TIF) [file pone.0279261.s002.tif]

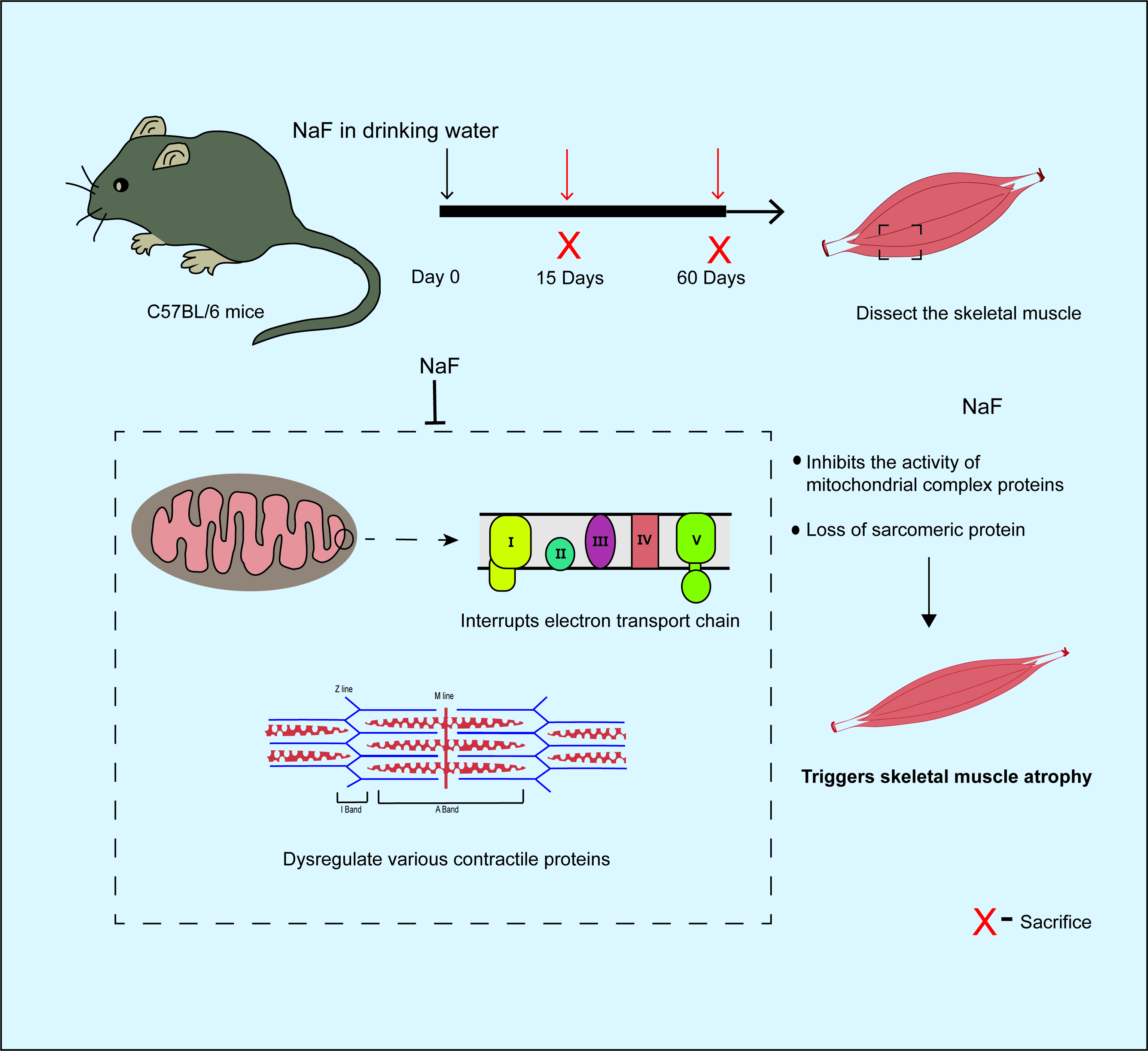

Supplement: S1 Graphical abstract — (TIF) [file pone.0279261.s007.tif]
