# Supplementary material for: Sodium fluoride induces skeletal muscle atrophy via changes in mitochondrial and sarcomeric proteomes
Source: PLoS One. 2022 Dec 22;17(12):e0279261. doi: 10.1371/journal.pone.0279261 (PMC9779014; doi:10.1371/journal.pone.0279261)
Supplement: S1 Table — (DOCX) [file pone.0279261.s003.docx]

**Table 1: Detail of primers that are utilized for the study**

| **Primer Name** | **Forward sequence** | **Reverse sequence** |
| --- | --- | --- |
| Ndufs1 | GAGAGCCGGCAGCCATCAT | TTACTTGCTGCTGTGCCAGT |
| Ndufs3 | CGGAGTGATGTGACCCACAA | TCAAGGCAGGACACCTGAAC |
| Ndufs4 | GTTGGGCATCAACCGCTGAC | GACTTGGACTTGGGTTTCGGA |
| Trim-54 | GTGCCAGACCATTGAGGACA | CTTGAAGCAGTTCGCCCTTG |
| NEDD8 | CGAACCCACAGACAAGGTGGA | TTCTTCACTGCCCAAGACCAC |
| Ankyrin 1 | GCTGGTGCTGTTAGGCTTCT | AAGCTCATCCCCCTTCAGGA |
| PSMA4 | CCGTCTTCCTCTGGAATCAAC | CTTGTGGATGTTGCGCCTCT |
| PSMB3 | CTGACGTCCAGACAGTTGCC | AAGGGCTTGAAGGTCTTCGG |
| PSMB9 | AAGTCCACACCGGGACAAC | GAGGGGAGAGCTTGTCGAAC |
| PSMC5 | CATCGCACAGCCAAAGGGAG | TGACAAACAGCTCCCTCACC |
| PSMD11 | GAACTGGGTTCTCTCCTGGC | CATAGCTCGACCTCCTGCC |
| PSMG3 | TTTCGGTCCCAACTGCTGTC | TTCTTCAGCGGAGGCGGG |
| PSMD9 | GGCACAACATCATCTGTCTCCA | GTTCATGGCCTCTTCTCGGG |
| Cullin 5 | TCTGTGGAAGCACTCATGGA | CCAAGTCCTTCAACATCGGC |
| Asb2 | TCATGCGGTGGGCCAGA | GCAGGTGACAAATCCCTGCAA |
| Ubd | TCTGTCCGCACCTGTGTTGT | GGGGCTTGAGGATTTTGGAGT |
| GAPDH | GTCGGTGTGAACGGATTTG | AATTTGCCGTGAGTGGAGTC |

|  |  |
| --- | --- |
|  |  |
|  |  |
|  |  |
|  |  |
